# Measurement of Pulsatile Insulin Secretion: Rationale and Methodology

**DOI:** 10.3390/metabo11070409

**Published:** 2021-06-22

**Authors:** Marcello C. Laurenti, Aleksey Matveyenko, Adrian Vella

**Affiliations:** 1 Division of Endocrinology, Diabetes & Metabolism, Mayo Clinic, Rochester, MN 55905, USA; laurenti.marcello@mayo.edu (M.C.L.); matveyenko.aleksey@mayo.edu (A.M.); 2 Biomedical Engineering and Physiology Graduate Program, Mayo Clinic Graduate School of Biomedical Sciences, Rochester, MN 55905, USA

**Keywords:** insulin pulses, C-peptide kinetics, hormone deconvolution, pulsatile insulin secretion

## Abstract

Pancreatic β-cells are responsible for the synthesis and exocytosis of insulin in response to an increase in circulating glucose. Insulin secretion occurs in a pulsatile manner, with oscillatory pulses superimposed on a basal secretion rate. Insulin pulses are a marker of β-cell health, and secretory parameters, such as pulse amplitude, time interval and frequency distribution, are impaired in obesity, aging and type 2 diabetes. In this review, we detail the mechanisms of insulin production and β-cell synchronization that regulate pulsatile insulin secretion, and we discuss the challenges to consider when measuring fast oscillatory secretion in vivo. These include the anatomical difficulties of measuring portal vein insulin noninvasively in humans before the hormone is extracted by the liver and quickly removed from the circulation. Peripheral concentrations of insulin or C-peptide, a peptide cosecreted with insulin, can be used to estimate their secretion profile, but mathematical deconvolution is required. Parametric and nonparametric approaches to the deconvolution problem are evaluated, alongside the assumptions and trade-offs required for their application in the quantification of unknown insulin secretory rates from known peripheral concentrations. Finally, we discuss the therapeutical implication of targeting impaired pulsatile secretion and its diagnostic value as an early indicator of β-cell stress.

## 1. Introduction

Insulin is a key hormone in glucose regulation, as its primary action is to stimulate glucose uptake and suppress glucose production. In response to increased blood glucose, following meal ingestion, insulin concentrations rise within minutes. In the opposite scenario, if plasma glucose decreases below 60 mg/dL, plasma insulin concentrations decrease by 80–90% [1]. The circulating concentration of insulin is, therefore, tightly regulated by the only cell type able to synthesize and secrete insulin: the pancreatic β-cell.

One of the most fascinating aspects of these unique cells, like many other endocrine glands [2], is that their secretion is characterized by discrete bursts or pulses, which can be used as a marker of β-cell synchronization and health [3,4]. The aim of this review is to summarize current knowledge of the mechanism(s) underlying pulsatile insulin secretion and review methods for its quantification in humans. Furthermore, we detail the implications of dysregulated pulsatile secretion and its role in the progression of β-cell failure.

### 1.1. Insulin Physiology

β-cells are located within the islets of Langerhans, where they are the predominant cell type in a cluster of endocrine cells that includes α-cells, responsible for glucagon production and secretion; δ-cells, responsible for somatostatin regulation; and PP cells, which produce pancreatic polypeptide [5]. The islets of Langerhans have a unique cytoarchitecture, which is species-dependent [6] and reflects its overall function [7]. This conformation facilitates autocrine and paracrine signaling, which coordinates the β-cells and their insulin secretion through the presence of Connexin36 (Cx36) gap-junctions [8], and both glucagon and somatostatin receptors [9,10]. Indeed, insulin secretion is impaired when β-cells are isolated from islets or when glucagon receptors are deleted [11,12,13].

Insulin production starts with the transcription of the INS gene located on chromosome 11 [14]. Transcriptional regulation of this gene is complex and remains an area of active research, but it seems to be controlled by at least three transcription factors, Pdx-1, NeuroD1 and MafA [15], in response to increased glucose concentrations and repressed following exposure to diabetogenic stressors such as proinflammatory cytokines (e.g., IL-1β) and saturated free fatty acids (e.g., palmitate). This mRNA sequence is then converted into preproinsulin, a precursor (110 amino acids) of insulin; translocated towards the ER membrane; folded; and transported to the Golgi [16,17].

The trans-Golgi network is the site where proinsulin is packed into insulin secretory granules (ISGs) and matures through a condensation process that occurs over a 3 h period [18]. This maturation is controlled by the prohormone converting enzyme PC1/3 [19], whose activity is increased by the acidification of the ISG lumen, achieved by the vesicular ATP-dependent proton pump (V-ATPase). It is important to note that the endpoint of this process is to convert proinsulin into its insulin hormone and an inert fragment known as C-peptide [19]. The Golgi apparatus also directs ISG trafficking, moving the mature granule toward one of two insulin granule pools [20]: the inner reserve pool and the readily releasable pool, which comprises all granules in close proximity to the cell membrane. Periodic and coordinated secretion of groups of granules is what gives rise to a burst of insulin and, ultimately, to pulsatile secretion. 

### 1.2. Mechanisms of Pulsatile Insulin Secretion

In β-cells, the signal for insulin exocytosis starts with an increase in glucose uptake from the cell. Glucose phosphorylation leads to pyruvate production, which enters the mitochondria to generate ATP. This allows the closure of ATP-sensitive K^+^ channels, resulting in membrane depolarization and Ca^2+^ influx, which triggers the release of insulin granules [21]. While glucose is considered the main stimulus to control insulin secretion, other nutrients such as fatty acids and amino acids can also stimulate granule exocytosis [22]. In response to this complex interplay of stimuli, the β-cells respond with the docking of the readily releasable granules to the membrane, their priming and finally their fusion. This constitutes the exocytosis of the first phase of insulin secretion [23], while the cell also recruits the granules in the reserve pool, transported by microtubules and F-actin tracks towards the membrane [24], to either replenish the readily releasable pool or to sustain prolonged secretion (second phase).

It is important to note that a single granule in a human β-cell contains about 1.7 amol of insulin and that a single β-cell stores about 10^4^ granules [25]. Even if this single β-cell secreted all its insulin granules simultaneously, the amount of insulin released into the circulation would not be discernable as a pulse in the peripheral circulation. The ability to observe an oscillatory pattern is due to the fact that pancreatic islets are capable of intra- and inter-islet synchronization [3]. While the mechanism underlying a coordinate, simultaneous secretion of insulin from multiple β-cells is not completely understood, different hypotheses have been formulated over the years.

As previously discussed, isolated islet cells show a lower response to a glucose stimulus [11], implying that β–β cell contacts are important for a sustained, coordinated secretion. Indeed, one of the possible reasons for pulsatile secretion could be electrical coupling between different cells, where raising intracellular levels of Ca^2+^, flowing through gap junctions, can cause simultaneous exocytosis in multiple β-cells [8,26]. This idea, together with multiple sources of experimental data, led to the development of the dual oscillator model, which assumes a Ca^2+^-dependent coordination of fast electrical bursts in β-cells, plus a glycolytic-dependent coordination of slow bursts in β-cells [27]. However, to explain the complexity of the synchronization necessary for physiologic insulin secretion, other possible mechanisms of β-cell coordination have been proposed. Some authors suggest that the islets, innervated by central nerves, are synchronized directly by cholinergic stimulation [28,29]. More recently, the presence of pacemaker-like β-cells (hubs) has been also proposed [30]. According to this theory, hub cells exhibit the ability to dictate the entrainment of their neighboring cells and initiate the electrical activity and Ca^2+^ oscillations in the β-cell’s cytosol [31]. However, the nature of the mechanism that hub cells would use to synchronize the activity of the rest of the islet is unclear. Indeed, a recent analysis [32] excludes the possibility that hub cells operate via electrical coupling and suggests instead that the key to the synchronization of β-cells could reside in a diffusive paracrine signal, such as nitric oxide, carbon monoxide or GABA [33,34,35]. 

### 1.3. Insulin Metabolism

After its release into the hepatic portal vein, the liver is the first organ encountered by insulin. This organ plays an important role in the systemic appearance of insulin, as the liver itself accounts for the majority of the insulin uptake and degradation (40–80%). This high percentage of extraction seems to be proportional to insulin pulse mass, and its amount decreases with defects in insulin secretion [36,37]. At its base physiological level, insulin clearance by the liver is a complex phenomenon that has not been completely decoded and requires a combination of passive and active processes. Extraction starts with the ability of insulin to cross the liver sinusoidal endothelial cells (LSECs) thanks to the presence of transcellular pores called fenestrations [38]. Subsequently, the activation of the insulin receptor tyrosine kinase in the hepatocyte and the phosphorylation of the glycoprotein CEACAM1 promote receptor-mediated insulin uptake into the hepatocyte, responsible for insulin degradation [39]. 

After its passage through the liver, insulin is diluted in the hepatic vein and, subsequently, the systemic insulin pool [40,41]. In one of the few experiments that performed portal vein sampling in humans, Song et al. showed [42] that insulin pulse amplitude decreased 5-fold from the portal vein to the peripheral circulation. A similar trend was also visible in our own experiments, from the hepatic vein to the periphery [43], suggesting that both hepatic extraction and the short half-life of insulin in the circulation (4–8 min [41]) can explain the substantially lower systemic concentrations.

Following its appearance in the circulation, insulin regulates glucose metabolism through positive and negative physiological feedback. Insulin receptors are present not only in the liver, as already mentioned, but also in the kidneys, skeletal muscle, adipose tissue and central nervous system (CNS). This activates different pathways and responses: insulin suppresses glucose production from the liver and the kidneys [44,45], increases glucose utilization by the insulin-dependent organs (skeletal muscle and adipose tissue [46]), promotes glycogen synthesis by the liver [47] and regulates feeding behavior through its actions in the CNS [48].

### 1.4. Characteristics of the Pulsatile Secretion Signal

So far, we have described the production and secretion of insulin by the pancreatic β-cells, as well as possible mechanisms that coordinate a synchronized secretion response. Before we overview how this physiological signal can be measured, it is important to formalize some definitions. Pulsatile secretion is used to define the quick and periodic release of a hormone over time. The signal is characterized by an oscillatory behavior, where multiple, consecutive pulses oscillate around a stable baseline. Finally, a pulse (or secretory burst) is defined as the rapid increase and subsequent decrease in the measured output.

**Figure 1:**
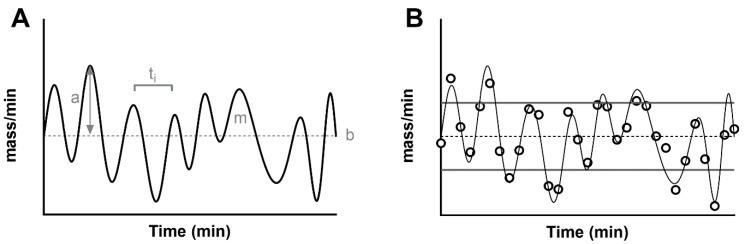
(**A**) Simulated, noise-free, pulsatile insulin secretion (thick black line). The signal is overlapped to its average (basal secretion, ‘b’, thin dashed line). The maximum value from the basal indicates the amplitude of the pulse (gray arrow, ‘a’), while the area under each pulse indicates its mass (‘m’). The distance between two pulses indicates the time interval (t_i_). (**B**) The panel shows a noisy realization of the secretion (black dots), sampled from the noise-free signal (thin black line). In addition to the basal secretion (thin dashed lines), the figure shows the standard deviation of the secretion as a measurement of pulse amplitude (basal ± SD, thick gray lines).

A simulated secretion profile, created with methodologies similar to those in [49], is shown in Figure 1A, which helps us to highlight some of the features of the signal. First of all, a secretion rate records the release of a mass over time, (i.e., mass/min, *Y*-axis). The average secretion in the time window is called the basal secretion. For simplicity, this is usually assumed to be time-invariant in the time windows under exam. In reality, basal secretion is the ultimate result of insulin production rate and clearance, and its set point fluctuates over time, affected by multiple factors such as glycemic state, physical exercise and sleep. For this reason, the common assumption of a time-invariant basal secretion is a true approximation only in short time windows, while it is known that the amount of insulin secretion changes as a result of ultradian and circadian rhythms [50].

In general, a series of pulses oscillate around the basal, and they can be characterized by an amplitude and a mass. Some methodologies described below assume that pulses strictly arise from the constant basal. With this modelization, the amplitude is always a positive value. By using a more general definition of a stationary signal (a signal whose parameters such as mean and variance remain constant over time), the constant mean of the signal in the time window becomes the measurement of the basal secretion, and the pulses oscillate around it. By definition, a measure such as the standard deviation of the signal can be used to characterize the average variability of fluctuations (pulse mass) above and below the basal (Figure 1B). Similarly, the mass secreted can be calculated as the area under the curve of each pulse.

Finally, another important parameter of interest is the time interval between two consecutive pulses. The measurement of this parameter has been a source of discussion since its early measurement in humans. Early experiments estimated a time interval of about 15 min [51], while more recent studies agree on a shorter period of about 5 min [4,52]. The heterogeneity of those values is the result of multiple factors that impact the estimation of the time interval between pulses. First of all, this parameter is strongly limited by the sampling schedule, and, in the ideal scenario, one can only measure a distance between two pulses that is double the sampling frequency. For example, if we expect to have a pulse every 4 min, we will need to collect a blood sample at a minimum of every 2 min (Figure 1B). Another limitation is the analytical technique used to measure the peptide in the blood samples. An assay with an elevated measurement error can dampen true pulses and create noisy oscillations. For these reasons, multiple methodologies have been employed to detect a pulse in a noisy signal ranging from early manual inspection of the signal [53] to algorithms that link the increase of the signal (due to a pulse) to some objective threshold such as the assay error or the spectral density of the signal [4,54,55,56]. 

## 2. Estimation of Secretion: The Deconvolution Problem

The study of the secretion rates of a hormone (i.e., mol/min) is a complex problem, because the production and secretion of a substrate are not often measurable directly in vivo. What is generally available is the causally related effect of that secretion on the circulation, by measuring the concentration of the hormone (i.e., mol/L). In a linear time-invariant (LTI) system, this cause–effect relationship can be expressed as a convolution integral:

(1) $$C\left(t\right)=\int_{-\infty }^{t}g\left(t-\tau \right)SR\left(\tau \right)d\tau$$

where C(t) is the plasmatic concentration of the substrate, g(t) is the impulse response of the system and *SR*(t) is the secretion rate of the substrate.

A simple dimensional analysis of Equation (1) will show that, in order to correctly solve the integral, the impulse response of the system needs to be in the function of a volume of distribution (i.e., 1/L). This is indeed the kinetic response of the substrate under analysis, generally expressed as a sum of exponentials.

Before we discuss how this problem can be solved, let us contextualize Equation (1) to our insulin secretion problem.

### 2.1. Choice of the Substrate to Measure

As we discussed above, we can interchangeably talk about insulin secretion and C-peptide secretion, given that those two peptides are produced in an equimolar ratio. There are, however, two main differences in their physiology that affect the concentration that we can measure in peripheral circulation.

First of all, we have seen that one of the most essential elements in the deconvolution problem is the kinetic response of the substrate. On this side, insulin shows a simpler single-exponential response with a fast half-life of ~6 min [41,53,57], while C-peptide shows a typical double-exponential response with a longer half-life (~35 min) [58,59]. More importantly, after its secretion, insulin undergoes a variable but significant (40–80%) hepatic extraction before it arrives into the circulation [36], while C-peptide is unaffected by its passage through the liver [41]. 

**Figure 2:**
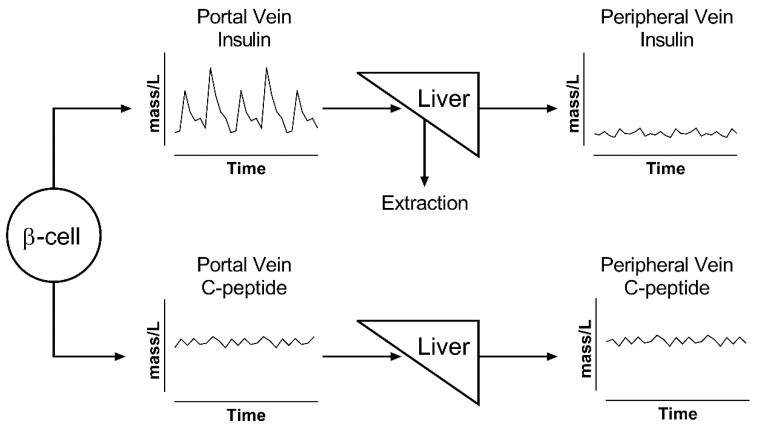
Illustration of changes in pulse oscillation for insulin (top) and C-peptide (bottom). β-cells secrete both peptides in equimolar ratio into the portal vein. Insulin has a rapid kinetics and is extracted by the liver; thus, its peripheral levels show both reduced concentrations and lower pulse amplitude. All of the released C-peptide reaches the periphery and accumulates in the circulation; thus, its portal concentrations are similar to those in the periphery.

The effects of these considerations are summarized in Figure 2, which shows that, while insulin and C-peptide are cosecreted by β-cells, their portal vein concentrations are drastically different. Portal vein concentrations of insulin show a very pulsatile signal, which is attenuated after its passage through the liver, because of nonlinear hepatic extraction. In contrast, portal and peripheral vein concentrations of C-peptide show a similar profile due to its negligible extraction. C-peptide has higher concentration values and a lower signal-to-noise ratio for pulse identification due to its longer half-life, which acts as a low-pass filter that decreases the signal-to-noise ratio of oscillations and makes C-peptide accumulate in the circulation [36,40,42,52].

Taken together, these two effects imply that the use of C-peptide is a better option for the study of total insulin secretion but makes the study of pulsatile secretion more challenging.

For this reason, multiple investigators chose to use insulin concentration as the main substrate for their analysis [60,61,62,63]. However, their analyses only reflect hepatic vein pulsatile secretion rates, not portal vein rates. Given the nonlinear and pulse-dependent hepatic extraction [36], the two rates above could be significantly different. 

On the other hand, C-peptide has been widely used to estimate total insulin secretion [60,64,65], but its use in pulse investigation is historically limited [66]. To overcome the challenge imposed by the longer half-life of C-peptide, we recently proposed a new methodology [43] that combines nonparametric deconvolution with a high sampling frequency of peripheral vein blood. Our in silico simulations showed that a very precise C-peptide assay (coefficient of variation of around 1%) is needed to correctly estimate pulsatile secretion from this substrate.

### 2.2. Choice of the Deconvolution Methodology

Once we have chosen the substrate to measure and we know its kinetic parameters, the formulation of the problem in Equation (1) allows us to relate the measurand (C-peptide or insulin) with the amount of insulin produced, after its modulation given by the systemic response. In other words, when the plasma concentration of the substrate and its kinetic parameters are available, the secretion rate becomes the solution of a deconvolution problem. Deconvolution is an inverse problem that presents some known challenges, identified in detail in [67]. Briefly, the problem is ill-posed and ill-conditioned [68], where the first definition implies that the finite number of observations does not permit a unique solution for the secretion, while the latter implies that a small percentage error in the measured concentration can produce a much greater percentage error in the reconstructed secretion, meaning that the estimation process is very sensitive to measurement error. 

#### 2.2.1. Parametric Deconvolution

The parametric solution of the deconvolution problem requires a strong assumption *a priori*, which answers the question: what is the shape of a secretory burst? If we assume that different secretory events from the same gland are homogeneous over time, they can be described with an algebraic formula. In this way, the secretion estimation problem is shifted to a parameter estimation problem. Veldhuis et al. pioneered this parametric approach, suggesting a mathematical description of a pulse, from a simple impulse function to a more complex waveform, including a Gaussian, square or gamma function [2,69]. While the exact shape of the secretory burst is not always known, its choice is ultimately a compromise between the *a priori* physiological signal, deduced by sampling the blood concentration of the substrate, and the mathematical complexity of the problem. Indeed, the more complex the secretory waveform, the more parameters need to be estimated.

In the case of insulin, researchers have successfully used this method [40,52,60,62,63] by assuming that a secretory burst is a Gaussian pulse that rises from a basal time-invariant secretory rate. The substrate secretion can then be described as:

(2) $$\mathrm{Sec}\mathrm{retion}\left(t\right)=\overset{n}{\underset{i=1}{\sum }}A_{i}{\;e}^{-\frac{1}{2}\;\frac{{\left(t_{i}-t\right)}^{2}}{{\mathrm{SD}}^{2}}}$$

where A_i_ is the amplitude of the ith secretory event occurring at time t_i_ and SD represents the standard deviation of the pulse, proportional to its half-duration. A further simplification of the problem is commonly used, which is to assume that different pulses can have individual values and amplitudes but a common half-duration.

**Figure 3:**
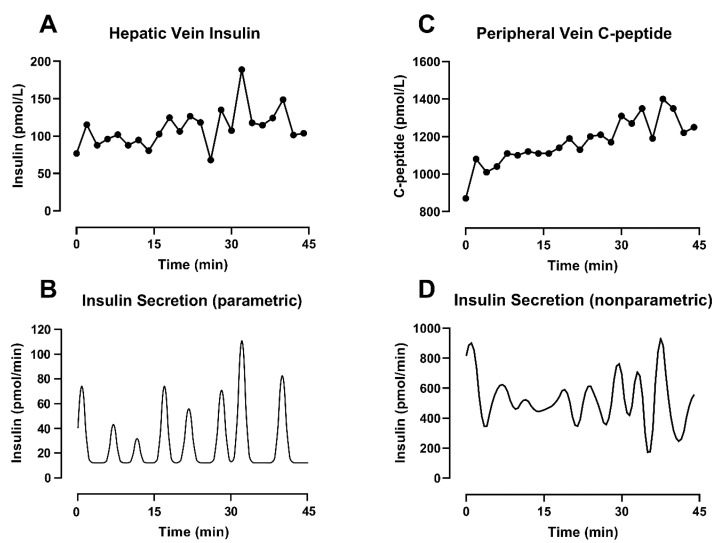
Different deconvolution approaches applied on different substrates measured from the same subjects during a hyperglycemic clamp started at t = −30 min. (**A**) Insulin concentration sampled every 2 min from the hepatic vein. (**B**) Insulin secretion estimated by parametric deconvolution applied on hepatic vein insulin concentration (**C**) C-peptide concentration sampled every 2 min from the peripheral vein. (**D**) Insulin secretion estimated by nonparametric deconvolution applied on peripheral vein C-peptide concentration.

With this approach, the secretion estimation is reduced to the identification of three elements: the (constant) basal secretion rate, the location in time of the individual pulses and the amplitude of the individual pulses [70]. The final secretion profile is the result of an iterative evaluation of the integral of Equation (1), which provides an estimated concentration. To solve the formula, the kinetics of the substrate are known and assumed to be time-invariant, while the parameters of the secretion profile can change in order to ensure the goodness of fit and minimize the difference between the measured concentration and its estimation. An example of this methodology can be seen in Figure 3A,B, where parametric deconvolution is applied to insulin concentration collected from the hepatic vein during a hyperglycemic clamp.

In conclusion, a parametric deconvolution approach comes with the innate necessity of formulating strong physiology-based assumptions, but it has the advantage of providing a regular, positive-only secretion profile for the hormone or substrate under analysis. 

#### 2.2.2. Nonparametric Deconvolution

The opposite of the parametric approach, which requires a known algebraical expression depending on a small number of unknown parameters, is the nonparametric approach which does not make assumptions on the shape of the solution and is adaptable to a wide range of applications. The deconvolution problem can then be solved by reverting to regularization methodologies, such as the Philips–Tikhonov regularization [71,72]. In its intuitive formulation, this approach uses the available concentration data to estimate the secretion rate that would have generated them. Furthermore, to reduce the effect of noisy concentration measurements, which would amplify the noise on the secretion, the method introduces a constraint on the regularity of the solution to penalize over-oscillating signals.

Briefly, the continuous integral of Equation (1) can be discretized to include only the sampled noisy observations of measurand, available on the uniform sampling grid.

(3) $$C\left(t_{k}\right)=\overset{k}{\underset{i=1}{\sum }}{\mathrm{SR}}_{i}\int_{t_{i}-1}^{t_{i}}g\left(t_{k}-\tau \right)d\tau =\overset{k}{\underset{i=1}{\sum }}{\mathrm{SR}}_{i}g_{k,i}$$

where SR*_i_* can now be interpreted as the mean level of secretion rate in the *i*th sampling interval. Adopting a more compact matrix notation and assuming the measurement error to be additive, Equation (3) becomes:

(4) y=Gu+v

where *y* is the n-dimensional vector containing the measured concentration, *u* denotes the secretion input of the system (the n-dimensional vector whose component are samples of the secretion rate), *v* is the n-dimensional vector of the measurement error and *G* is an n × n lower triangular matrix containing the information on the impulse response g(t). 

By applying the Phillips–Tikhonov regularization, this problem can now be solved by estimating the secretion input that minimizes the objective function (OF):

(5) $$OF=argmin_{u}{\left(y-Gu\right)}^{T}\Sigma_{v}^{-1}\left(y-Gu\right)+\gamma u^{T}F^{T}Fu$$

Under this formulation, $\Sigma_{v}$ is an n × n diagonal matrix of the measurement error and, in the first term of the equation, helps to weight the fit of the data. The second term of the equation contributes to the smoothness. There, *F* is a square penalty matrix with the same dimension as *u* and contains the *m*th order difference vector of u. Finally, *γ* is a non-negative parameter referred to as a regularization parameter. The correct choice of *γ* is critical for reducing the effect of noise on the data, and it has been the focus of different studies [67,73,74]. Of interest is the method by De Nicolao et al. [67], which adopts a probabilistic description of the unknown secretion signal and suggests tuning the regularization parameter in order to maximize the likelihood of the measurements. An example of deconvolution using this methodology can be seen in Figure 3C,D, where nonparametric deconvolution was applied to the C-peptide concentrations observed in the peripheral circulation during a hyperglycemic clamp.

In conclusion, the nonparametric deconvolution problem can be solved by a minimization of an objective function that is a trade-off between a good data fit and a certain degree of smoothing. The method has the underlying advantage of decreasing the need for *a priori* assumptions of insulin or C-peptide physiology, but it could result in secretion profiles that are less regular than their parametric counterparts.

## 3. Summary and Future Directions

The study of pulsatile insulin secretion is a fascinating and challenging problem, due to both its physiology and the presence of high-frequency oscillations. The kinetic parameters of the peptide under study (volume of distribution, half-life and clearance), its hepatic clearance and pancreatic secretion into the portal vein (an inaccessible sampling site) are all factors that influence the shape of the concentration signal that we can measure in the periphery. 

Deconvolution is a useful mathematical tool that allows us to partially overcome those challenges by estimating an unknown insulin secretion from a measurable concentration. This method has been widely used to analyze pulsatile insulin release in the systemic circulation, but it is important to remember that its results are strongly affected by the sampling frequency, the choice of the substrate (C-peptide or insulin) and the *a priori* assumptions on the expected secretion signals. For this reason, it is always important to contextualize the conclusions of a study as a function of the methods used to estimate the secretory signal.

This is evident in Figure 3, bottom panels, where the combined effect of different substrates and methodologies leads to different secretion profiles, even when the concentration data are collected simultaneously in the same subject. The physiology can partially help to explain those differences. The basal secretion rate estimated from insulin concentrations (Figure 3B) is lower compared to the one obtained from C-peptide (Figure 3D). As discussed before, this is most likely due to nonlinear hepatic extraction (>50%) for insulin, as well as slower C-peptide clearance. The latter explains C-peptide accumulation in the circulation, especially in response to sustained β-cell activity such as during a hyperglycemic clamp (as used in our experiments). These differences clearly also affect the amplitude of the pulses. However, in this example, the two methodologies show a similar position in time for the pulses. This is in keeping with our previous simulation study [43] where we reported that errors in the assay or in the value of the kinetic parameters (individually measured or estimated using a population model) could affect the amplitude of individual pulses, but the positions in time of the peaks remain consistent despite additional sources of noise.

Despite these limitations, multiple independent studies agree on the physiological significance of the pulsatile release of insulin, advocating for the importance of its study. For example, some studies have shown that pulsatile insulin infusion has a greater ability to promote more efficient glucose utilization compared to a constant infusion [75], both during a constant glucose infusion [76] and during a meal-like infusion [77]. A similar result was observed on endogenous glucose production (EGP) during a euglycemic insulin clamp, when a pulsatile insulin profile required a 40% lower infusion rate to suppress EGP equally to that observed with a constant insulin infusion [78], and subsequently confirmed by animal experiments and mathematical models [52,79].

The studies described above used somatostatin to suppress endogenous insulin production and insulin infusion pumps to deliver exogenous insulin into the peripheral circulation. In the in vivo study of pulsatile insulin secretion, multiple studies utilized the deconvolution techniques described above. Despite the differences in approach, endogenous pulsatile insulin secretion has been generally considered a marker of β-cell health. This choice is justified by the fact that insulin pulses contribute 60 to 85% of the total insulin secretion [53], and changes to this pattern have been associated with insulin resistance and type 2 diabetes (T2D) [61,80,81]. In particular, it has been reported that not only T2D but also aging and obesity can cause a reduction in pulse amplitude and an increase in the time interval between pulses [82,83,84], with an increase in basal—rather than pulsatile—secretion possibly mediated by a chronic exposure to increased nutrients such as glucose or fatty acids [85,86]. Changes in pulsatile insulin secretion not only worsen the ability to regulate blood glucose [60] but also affect the hepatic metabolism. The liver is sensitive to rapid fluctuations in insulin concentration [36], and defects in secretion, such as the delivery of nonpulsatile insulin secretion, impair hepatic insulin signaling and action, although it is still not clear which feature of pulses measured in humans controls hepatic insulin sensitivity [52,87]. 

Furthermore, by empowering the use of approximate entropy [88], a statistical index that measures the randomness of a time series, independent analysis showed that pulsatile secretion profile is more regular in health and it tends to higher values of randomness in T2D [63,89] and in prediabetic subjects [4]. In addition, we have recently suggested that the average time interval between pulses, which provides an accessible number to describe the periodicity of the signal, might not be able to discern the contribution of different secretion rates, a consequence of different levels of islet coordination. We have therefore proposed that an index of pulse dispersion, such as the frequency dispersion index [90] measured on the Fourier transform of the secretion, could describe how the coordination of pancreatic islets changes in response to disease and to metabolic state. In our dataset [4], we reported that healthy subjects showed a greater ability to coordinate their secretion, compared to their fasting levels, in response to hyperglycemia. 

In conclusion, insulin pulsatility is an important characteristic of endogenous insulin secretion and β-cell health. Important downstream signaling pathways, such as glucose uptake and hepatic response to insulin, are optimal under conditions of intact pulsatility. An impairment or loss of pulsatility is an early marker of β-cell stress and overload [4,81] and in the future could be used as an early marker of β-cell failure. At least in the early stages, these defects seem to be reversible [60,91,92]. Targeting insulin pulsatility may have therapeutic benefit in prediabetic subjects. In vivo studies of pulsatility require a rapid sampling schedule and the use of deconvolution techniques to estimate an individualized secretion profile. These studies are, however, necessary to further understand the physiological effect of pulses and to develop approaches to restore pulsatile insulin secretion.

## References

[1] Woerle H.J., Meyer C., Dostou J.M., Gosmanov N.R., Islam N., Popa E., Wittlin S.D., Welle S.L., Gerich J.E. (2003). Pathways for glucose disposal after meal ingestion in humans. Am. J. Physiol. Endocrinol. Metab..

[2] Veldhuis J.D., Keenan D.M., Pincus S.M. (2008). Motivations and methods for analyzing pulsatile hormone secretion. Endocr. Rev..

[3] Pedersen M.G., Bertram R., Sherman A. (2005). Intra- and inter-islet synchronization of metabolically driven insulin secretion. Biophys. J..

[4] Laurenti M.C., Dalla Man C., Varghese R.T., Andrews J.C., Rizza R.A., Matveyenko A., De Nicolao G., Cobelli C., Vella A. (2020). Diabetes-associated genetic variation in TCF7L2 alters pulsatile insulin secretion in humans. JCI Insight.

[5] Da Silva Xavier G. (2018). The Cells of the Islets of Langerhans. J. Clin. Med..

[6] Cabrera O., Berman D.M., Kenyon N.S., Ricordi C., Berggren P.O., Caicedo A. (2006). The unique cytoarchitecture of human pancreatic islets has implications for islet cell function. Proc. Natl. Acad. Sci. USA.

[7] Kilimnik G., Zhao B., Jo J., Periwal V., Witkowski P., Misawa R., Hara M. (2011). Altered islet composition and disproportionate loss of large islets in patients with type 2 diabetes. PLoS ONE.

[8] Farnsworth N.L., Benninger R.K. (2014). New insights into the role of connexins in pancreatic islet function and diabetes. FEBS Lett..

[9] Wendt A., Eliasson L. (2020). Pancreatic alpha-cells-The unsung heroes in islet function. Semin. Cell Dev. Biol..

[10] Rodriguez-Diaz R., Tamayo A., Hara M., Caicedo A. (2020). The Local Paracrine Actions of the Pancreatic alpha-Cell. Diabetes.

[11] Ravier M.A., Guldenagel M., Charollais A., Gjinovci A., Caille D., Sohl G., Wollheim C.B., Willecke K., Henquin J.C., Meda P. (2005). Loss of connexin36 channels alters beta-cell coupling, islet synchronization of glucose-induced Ca2+ and insulin oscillations, and basal insulin release. Diabetes.

[12] Scarl R.T., Corbin K.L., Vann N.W., Smith H.M., Satin L.S., Sherman A., Nunemaker C.S. (2019). Intact pancreatic islets and dispersed beta-cells both generate intracellular calcium oscillations but differ in their responsiveness to glucose. Cell Calcium.

[13] Sorensen H., Winzell M.S., Brand C.L., Fosgerau K., Gelling R.W., Nishimura E., Ahren B. (2006). Glucagon receptor knockout mice display increased insulin sensitivity and impaired beta-cell function. Diabetes.

[14] Melloul D., Marshak S., Cerasi E. (2002). Regulation of insulin gene transcription. Diabetologia.

[15] Andrali S.S., Sampley M.L., Vanderford N.L., Ozcan S. (2008). Glucose regulation of insulin gene expression in pancreatic beta-cells. Biochem. J..

[16] Ghiasi S.M., Dahlby T., Hede Andersen C., Haataja L., Petersen S., Omar-Hmeadi M., Yang M., Pihl C., Bresson S.E., Khilji M.S. (2019). Endoplasmic Reticulum Chaperone Glucose-Regulated Protein 94 Is Essential for Proinsulin Handling. Diabetes.

[17] Greenman I.C., Gomez E., Moore C.E., Herbert T.P. (2005). The selective recruitment of mRNA to the ER and an increase in initiation are important for glucose-stimulated proinsulin synthesis in pancreatic beta-cells. Biochem. J..

[18] Arvan P., Halban P.A. (2004). Sorting ourselves out: Seeking consensus on trafficking in the beta-cell. Traffic.

[19] Ramzy A., Asadi A., Kieffer T.J. (2020). Revisiting Proinsulin Processing: Evidence That Human beta-Cells Process Proinsulin With Prohormone Convertase (PC) 1/3 but Not PC2. Diabetes.

[20] Bratanova-Tochkova T.K., Cheng H., Daniel S., Gunawardana S., Liu Y.J., Mulvaney-Musa J., Schermerhorn T., Straub S.G., Yajima H., Sharp G.W. (2002). Triggering and augmentation mechanisms, granule pools, and biphasic insulin secretion. Diabetes.

[21] McKenna J.P., Ha J., Merrins M.J., Satin L.S., Sherman A., Bertram R. (2016). Ca2+ Effects on ATP Production and Consumption Have Regulatory Roles on Oscillatory Islet Activity. Biophys. J..

[22] Newsholme P., Cruzat V., Arfuso F., Keane K. (2014). Nutrient regulation of insulin secretion and action. J. Endocrinol..

[23] Olofsson C.S., Gopel S.O., Barg S., Galvanovskis J., Ma X., Salehi A., Rorsman P., Eliasson L. (2002). Fast insulin secretion reflects exocytosis of docked granules in mouse pancreatic B-cells. Pflug. Arch..

[24] Zhu X., Hu R., Brissova M., Stein R.W., Powers A.C., Gu G., Kaverina I. (2015). Microtubules Negatively Regulate Insulin Secretion in Pancreatic beta Cells. Dev. Cell.

[25] Rorsman P., Renstrom E. (2003). Insulin granule dynamics in pancreatic beta cells. Diabetologia.

[26] Benninger R.K., Piston D.W. (2014). Cellular communication and heterogeneity in pancreatic islet insulin secretion dynamics. Trends Endocrinol. Metab..

[27] Bertram R., Satin L.S., Sherman A.S. (2018). Closing in on the Mechanisms of Pulsatile Insulin Secretion. Diabetes.

[28] Fendler B., Zhang M., Satin L., Bertram R. (2009). Synchronization of pancreatic islet oscillations by intrapancreatic ganglia: A modeling study. Biophys. J..

[29] Zhang M., Fendler B., Peercy B., Goel P., Bertram R., Sherman A., Satin L. (2008). Long lasting synchronization of calcium oscillations by cholinergic stimulation in isolated pancreatic islets. Biophys. J..

[30] Johnston N.R., Mitchell R.K., Haythorne E., Pessoa M.P., Semplici F., Ferrer J., Piemonti L., Marchetti P., Bugliani M., Bosco D. (2016). Beta Cell Hubs Dictate Pancreatic Islet Responses to Glucose. Cell Metab..

[31] Salem V., Silva L.D., Suba K., Georgiadou E., Neda Mousavy Gharavy S., Akhtar N., Martin-Alonso A., Gaboriau D.C.A., Rothery S.M., Stylianides T. (2019). Leader beta-cells coordinate Ca(2+) dynamics across pancreatic islets in vivo. Nat. Metab..

[32] Satin L.S., Zhang Q., Rorsman P. (2020). “Take Me To Your Leader”: An Electrophysiological Appraisal of the Role of Hub Cells in Pancreatic Islets. Diabetes.

[33] Gheibi S., Ghasemi A. (2020). Insulin secretion: The nitric oxide controversy. EXCLI J..

[34] Menegaz D., Hagan D.W., Almaca J., Cianciaruso C., Rodriguez-Diaz R., Molina J., Dolan R.M., Becker M.W., Schwalie P.C., Nano R. (2019). Mechanism and effects of pulsatile GABA secretion from cytosolic pools in the human beta cell. Nat. Metab..

[35] Rahman F.U., Park D.R., Joe Y., Jang K.Y., Chung H.T., Kim U.H. (2019). Critical Roles of Carbon Monoxide and Nitric Oxide in Ca(2+) Signaling for Insulin Secretion in Pancreatic Islets. Antioxid. Redox Signal..

[36] Meier J.J., Veldhuis J.D., Butler P.C. (2005). Pulsatile insulin secretion dictates systemic insulin delivery by regulating hepatic insulin extraction in humans. Diabetes.

[37] Sathananthan A., Dalla Man C., Zinsmeister A.R., Camilleri M., Rodeheffer R.J., Toffolo G., Cobelli C., Rizza R.A., Vella A. (2012). A concerted decline in insulin secretion and action occurs across the spectrum of fasting and postchallenge glucose concentrations. Clin. Endocrinol..

[38] Hunt N.J., Lockwood G.P., Warren A., Mao H., McCourt P.A.G., Le Couteur D.G., Cogger V.C. (2019). Manipulating fenestrations in young and old liver sinusoidal endothelial cells. Am. J. Physiol. Gastrointest. Liver Physiol..

[39] Najjar S.M., Perdomo G. (2019). Hepatic Insulin Clearance: Mechanism and Physiology. Physiology.

[40] Porksen N., Hollingdal M., Juhl C., Butler P., Veldhuis J.D., Schmitz O. (2002). Pulsatile insulin secretion: Detection, regulation, and role in diabetes. Diabetes.

[41] Piccinini F., Bergman R.N. (2020). The Measurement of Insulin Clearance. Diabetes Care.

[42] Song S.H., McIntyre S.S., Shah H., Veldhuis J.D., Hayes P.C., Butler P.C. (2000). Direct measurement of pulsatile insulin secretion from the portal vein in human subjects. J. Clin. Endocrinol. Metab..

[43] Laurenti M.C., Vella A., Varghese R.T., Andrews J.C., Sharma A., Kittah N.E., Rizza R.A., Matveyenko A., De Nicolao G., Cobelli C. (2019). Assessment of pulsatile insulin secretion derived from peripheral plasma C-peptide concentrations by nonparametric stochastic deconvolution. Am. J. Physiol. Endocrinol. Metab..

[44] Kaneko K., Soty M., Zitoun C., Duchampt A., Silva M., Philippe E., Gautier-Stein A., Rajas F., Mithieux G. (2018). The role of kidney in the inter-organ coordination of endogenous glucose production during fasting. Mol. Metab..

[45] Lewis G.F., Carpentier A.C., Pereira S., Hahn M., Giacca A. (2021). Direct and indirect control of hepatic glucose production by insulin. Cell Metab..

[46] Dimitriadis G., Mitrou P., Lambadiari V., Maratou E., Raptis S.A. (2011). Insulin effects in muscle and adipose tissue. Diabetes Res. Clin. Pract..

[47] Nozaki Y., Petersen M.C., Zhang D., Vatner D.F., Perry R.J., Abulizi A., Haedersdal S., Zhang X.-M., Butrico G.M., Samuel V.T. (2020). Metabolic control analysis of hepatic glycogen synthesis in vivo. Proc. Natl. Acad. Sci. USA.

[48] Mitchell C.S., Begg D.P. (2021). The regulation of food intake by insulin in the central nervous system. J. Neuroendocrinol..

[49] Sparacino G., Cobelli C. (1997). Impulse response model in reconstruction of insulin secretion by deconvolution: Role of input design in the identification experiment. Ann. Biomed. Eng..

[50] Simon C., Brandenberger G. (2002). Ultradian oscillations of insulin secretion in humans. Diabetes.

[51] Lang D.A., Matthews D.R., Peto J., Turner R.C. (1979). Cyclic oscillations of basal plasma glucose and insulin concentrations in human beings. N. Engl. J. Med..

[52] Matveyenko A.V., Liuwantara D., Gurlo T., Kirakossian D., Dalla Man C., Cobelli C., White M.F., Copps K.D., Volpi E., Fujita S. (2012). Pulsatile portal vein insulin delivery enhances hepatic insulin action and signaling. Diabetes.

[53] Porksen N., Nyholm B., Veldhuis J.D., Butler P.C., Schmitz O. (1997). In humans at least 75% of insulin secretion arises from punctuated insulin secretory bursts. Am. J. Physiol..

[54] Kushler R.H., Brown M.B. (1991). A model for the identification of hormone pulses. Stat. Med..

[55] Vidal A., Zhang Q., Medigue C., Fabre S., Clement F. (2012). DynPeak: An algorithm for pulse detection and frequency analysis in hormonal time series. PLoS ONE.

[56] Horton K.W., Carlson N.E., Grunwald G.K., Mulvahill M.J., Polotsky A.J. (2017). A population-based approach to analyzing pulses in time series of hormone data. Stat. Med..

[57] Campioni M., Toffolo G., Basu R., Rizza R.A., Cobelli C. (2009). Minimal model assessment of hepatic insulin extraction during an oral test from standard insulin kinetic parameters. Am. J. Physiol. Endocrinol. Metab..

[58] Van Cauter E., Mestrez F., Sturis J., Polonsky K.S. (1992). Estimation of insulin secretion rates from C-peptide levels. Comparison of individual and standard kinetic parameters for C-peptide clearance. Diabetes.

[59] Varghese R.T., Dalla Man C., Laurenti M.C., Piccinini F., Sharma A., Shah M., Bailey K.R., Rizza R.A., Cobelli C., Vella A. (2018). Performance of individually measured vs population-based C-peptide kinetics to assess beta-cell function in the presence and absence of acute insulin resistance. Diabetes Obes. Metab..

[60] Laedtke T., Kjems L., Porksen N., Schmitz O., Veldhuis J., Kao P.C., Butler P.C. (2000). Overnight inhibition of insulin secretion restores pulsatility and proinsulin/insulin ratio in type 2 diabetes. Am. J. Physiol. Endocrinol. Metab..

[61] Porksen N. (2002). Early changes in beta-cell function and insulin pulsatility as predictors for type 2 diabetes. Diabetes Nutr. Metab..

[62] Meier J.J., Pennartz C., Schenker N., Menge B.A., Schmidt W.E., Heise T., Kapitza C., Veldhuis J.D. (2013). Hyperglycaemia is associated with impaired pulsatile insulin secretion: Effect of basal insulin therapy. Diabetes Obes. Metab..

[63] Meneilly G.S., Veldhuis J.D., Elahi D. (2005). Deconvolution analysis of rapid insulin pulses before and after six weeks of continuous subcutaneous administration of glucagon-like peptide-1 in elderly patients with type 2 diabetes. J. Clin. Endocrinol. Metab..

[64] Sparacino G., Cobelli C. (1996). A stochastic deconvolution method to reconstruct insulin secretion rate after a glucose stimulus. IEEE Trans. Biomed. Eng..

[65] Cobelli C., Dalla Man C., Toffolo G., Basu R., Vella A., Rizza R. (2014). The oral minimal model method. Diabetes.

[66] Balks H.J., Schmidt A., Prank K., Hemmer F., von zur Muhlen A., Brabant G. (1992). Temporal pattern of pancreatic insulin and C-peptide secretion and of plasma glucose levels after nutritional stimulation. J. Clin. Endocrinol. Metab..

[67] De Nicolao G., Sparacino G., Cobelli C. (1997). Nonparametric input estimation in physiological systems: Problems, methods, and case studies. Automatica.

[68] Bertero M., Hawkes P.W. (1989). Linear Inverse and III-Posed Problems. Advances in Electronics and Electron Physics.

[69] Carlson N.E., Johnson T.D., Brown M.B. (2009). A Bayesian approach to modeling associations between pulsatile hormones. Biometrics.

[70] Johnson M.L., Pipes L., Veldhuis P.P., Farhy L.S., Boyd D.G., Evans W.S. (2008). AutoDecon, a deconvolution algorithm for identification and characterization of luteinizing hormone secretory bursts: Description and validation using synthetic data. Anal. Biochem..

[71] Phillips D.L. (1962). A Technique for the Numerical Solution of Certain Integral Equations of the First Kind. J. ACM.

[72] Tikhonov A. (1963). Solution of Incorrectly Formulated Problems and the Regularization Method. Sov. Math..

[73] Krawczyk-Stańdo D., Rudnicki M. (2007). Regularization Parameter Selection in Discrete Ill-Posed Problems—The Use of the U-Curve. Int. J. Appl. Math. Comput. Sci..

[74] Karoui A., Bear L., Migerditichan P., Zemzemi N. (2018). Evaluation of Fifteen Algorithms for the Resolution of the Electrocardiography Imaging Inverse Problem Using ex-vivo and in-silico Data. Front. Physiol..

[75] Matthews D.R., Naylor B.A., Jones R.G., Ward G.M., Turner R.C. (1983). Pulsatile insulin has greater hypoglycemic effect than continuous delivery. Diabetes.

[76] Sturis J., Scheen A.J., Leproult R., Polonsky K.S., van Cauter E. (1995). 24-hour glucose profiles during continuous or oscillatory insulin infusion. Demonstration of the functional significance of ultradian insulin oscillations. J. Clin. Investig..

[77] Juhl C.B., Gjedsted J., Nielsen M.F., Schmitz O. (2012). Increased action of pulsatile compared to non-pulsatile insulin delivery during a meal-like glucose exposure simulated by computerized infusion in healthy humans. Metabolism.

[78] Komjati M., Bratusch-Marrain P., Waldhausl W. (1986). Superior efficacy of pulsatile versus continuous hormone exposure on hepatic glucose production in vitro. Endocrinology.

[79] Zhao G., Wirth D., Schmitz I., Meyer-Hermann M. (2017). A mathematical model of the impact of insulin secretion dynamics on selective hepatic insulin resistance. Nat. Commun..

[80] Satin L.S., Butler P.C., Ha J., Sherman A.S. (2015). Pulsatile insulin secretion, impaired glucose tolerance and type 2 diabetes. Mol. Aspects Med..

[81] Schofield C.J., Sutherland C. (2012). Disordered insulin secretion in the development of insulin resistance and Type 2 diabetes. Diabet. Med..

[82] Goodner C.J., Koerker D.J., Weigle D.S., McCulloch D.K. (1989). Decreased insulin- and glucagon-pulse amplitude accompanying beta-cell deficiency induced by streptozocin in baboons. Diabetes.

[83] O’Rahilly S., Turner R.C., Matthews D.R. (1988). Impaired pulsatile secretion of insulin in relatives of patients with non-insulin-dependent diabetes. N. Engl. J. Med..

[84] Westacott M.J., Farnsworth N.L., St Clair J.R., Poffenberger G., Heintz A., Ludin N.W., Hart N.J., Powers A.C., Benninger R.K.P. (2017). Age-Dependent Decline in the Coordinated [Ca(2+)] and Insulin Secretory Dynamics in Human Pancreatic Islets. Diabetes.

[85] Erion K., Corkey B.E. (2018). beta-Cell Failure or beta-Cell Abuse?. Front. Endocrinol..

[86] Corbin K.L., Waters C.D., Shaffer B.K., Verrilli G.M., Nunemaker C.S. (2016). Islet Hypersensitivity to Glucose Is Associated With Disrupted Oscillations and Increased Impact of Proinflammatory Cytokines in Islets From Diabetes-Prone Male Mice. Endocrinology.

[87] Laurenti M.C., Dalla Man C., Varghese R.T., Andrews J.C., Jones J.G., Barosa C., Rizza R.A., Matveyenko A., De Nicolao G., Bailey K.R. (2021). Insulin Pulse Characteristics and Insulin Action in Non-diabetic Humans. J. Clin. Endocrinol. Metab..

[88] Pincus S. (1995). Approximate entropy (ApEn) as a complexity measure. Chaos.

[89] Menge B.A., Gruber L., Jorgensen S.M., Deacon C.F., Schmidt W.E., Veldhuis J.D., Holst J.J., Meier J.J. (2011). Loss of inverse relationship between pulsatile insulin and glucagon secretion in patients with type 2 diabetes. Diabetes.

[90] Prieto T.E., Myklebust J.B., Hoffmann R.G., Lovett E.G., Myklebust B.M. (1996). Measures of postural steadiness: Differences between healthy young and elderly adults. IEEE Trans. Biomed. Eng..

[91] Brown R.J., Rother K.I. (2008). Effects of beta-cell rest on beta-cell function: A review of clinical and preclinical data. Pediatr. Diabetes.

[92] Corezola do Amaral M.E., Kravets V., Dwulet J.M., Farnsworth N.L., Piscopio R., Schleicher W.E., Miranda J.G., Benninger R.K.P. (2020). Caloric restriction recovers impaired beta-cell-beta-cell gap junction coupling, calcium oscillation coordination, and insulin secretion in prediabetic mice. Am. J. Physiol. Endocrinol. Metab..

